# Early Assessment of Right Ventricular Function in Systemic Lupus Erythematosus Patients using Strain and Strain Rate Imaging

**DOI:** 10.5935/abc.20180091

**Published:** 2018-07

**Authors:** Runlan Luo, Hongyan Cui, Dongmei Huang, Lihua Sun, Shengda Song, Mengyao Sun, Guangsen Li

**Affiliations:** Department of Ultrasound, the Second Affiliated Hospital of Dalian Medical, Dalian, Liaoning - China

**Keywords:** Ventricular Function, Right / physiology, Lupus Erythematosus, Systemic, Hypertension, Pulmonary, Echocardiography

## Abstract

**Background:**

Right ventricular function is a crucial factor of the prognosis of systemic lupus erythematosus (SLE).

**Objectives:**

To evaluate the right ventricular function in SLE patients with different degrees of pulmonary hypertension (PH) by strain and strain rate imaging.

**Methods:**

A total of 102 SLE patients and 30 healthy volunteers were studied between October 2015 and May 2016. Patients were divided into three groups according to pulmonary artery systolic pressure (PASP) estimated by echocardiography: group control (A); PASP ≤ 30 mmHg (group B, n = 37); PASP 30-50 mmHg (mild PH; group C, n = 34); and PASP ≥ 50 mmHg (moderate-to-severe PH; group D, n = 31). Longitudinal peak systolic strain (ε) and strain rate (SR), including systolic strain rate (SRs), early diastolic strain rate (SRe) and late diastolic strain rate (SRa) were measured in the basal, middle and apical segments of the right ventricular free wall in participants by two-dimensional speckle tracking echocardiography (2D-STE) from the apical four-chamber view. A p < 0.05 was set for statistical significance.

**Results:**

The parameters of ε, SRs, SRe, and SRa were significantly decreased in groups C and D compared with groups A and B. The ε of each segments was significantly lower in group D than in group C, while there were no differences in SRs, SRe and SRa between groups C and D.

**Conclusions:**

Strain and strain rate imaging could early detect the right ventricular dysfunction in SLE patients with PH, and provide important value for clinical therapy and prognosis of these patients.

## Introduction

Systemic lupus erythematosus (SLE) is a chronic, autoimmune disorder involving multiple organs and systems, such as lung, muscle, skin, joint and heart, especially the right ventricle. Moreover, right ventricular (RV) function is a crucial factor for the prognosis of SLE patients.^1^ Pulmonary hypertension (PH) is a common, severe, and devastating complication of SLE, and its prevalence varies between 0.5 and 43%.^2^ It is an independent factor for SLE, with a 3-year survival rate of 44.9%.^3^ SLE combined with PH can cause RV dysfunction, and its mortality is closely related to the RV function.^4^ Thereby, early detection of subclinical RV dysfunction is important for the establishment of treatment strategy and improvement of prognosis in SLE patients with PH.

Although cardiac magnetic resonance and radionuclide angiography are considered gold standards for the assessment of RV systolic function, echocardiography is still widely used for its simplicity, low price, and non-invasiveness.^5^ However, assessment of the right ventricle is limited due to its thin wall and complex anatomy - a triangular shape from the lateral view, and a crescent shape from section view.^6^ It has been documented that two-dimensional speckle tracking echocardiography (2D-STE) derived strain and strain rate imaging, a novel technique with less dependence on the angle and intra/inter-observer variability, could reliably and qualitatively detect early subclinical RV dysfunction.^7-9^ In this study, strain refers in particular to the longitudinal peak systolic strain (ε), and represents the degree of myocardial deformation. Strain rate (SR) is the shortening velocity of the myocardium, *i.e.*, it represents the change in deformation over time.^10^ SR includes systolic SR (SRs), early diastolic SR (SRe) and late diastolic strain rate (SRa), which reflect cardiac contraction during systole and diastole, respectively.^11^

In this study, we aimed to assess the RV function through strain and SR by 2D-STE in SLE patients with PH estimated by echocardiography.

## Methods

### Study Subjects

A total of 102 SLE patients (M:F = 11:91; aged 20-52 years, mean age: 43.2 ± 9.3 years) and 30 age-matched healthy volunteers as control group (Group A) (M:F = 3:27, aged 23-51 years, mean age: 42.1 ± 10.5 years, mean pulmonary artery systolic pressure - PASP 22.54 ± 4.31 mmHg) were eligible to participate in this study. The study was conducted between October 2015 and May 2016 in our hospital. The eligibility criteria of SLE diagnosis met the 2012 Systemic Lupus International Collaborating Clinics (SLICC) standard criteria.^12^ Exclusion criteria included left ventricular heart failure, congenital heart diseases, coronary heart disease, cardiomyopathy and valvular heart disease, pericardial effusion, use of cardiotoxic drugs, history of hypertension, infectious myocarditis and pulmonary obstructive diseases. Eight patients with poor-quality echocardiographic imaging and ten patients unwilling to participate in the study were excluded.

The selected patients were divided into three groups according to the PASP estimated by echocardiography: Group B included 37 patients with PASP ≤ 30 mmHg, which was considered as a non-PH group (M:F = 4:33, aged 21-51 years, mean age 45.3 ± 8.4 years, mean PASP 23.61 ± 3.11 mmHg); Group C included 34 patients with 30 < PASP < 50 mmHg, considered as mild PH group, (M:F = 4:30, aged 20-52 years, mean age: 41.3 ± 9.6 years, mean PASP 45.11 ± 5.50 mmHg); and Group D included 31 patients with PASP ≥ 50 mmHg, which was considered as moderate to severe PH group (M:F = 3:28, aged 23-51 years, mean age: 43.3 ± 7.5 years, mean PASP: 72.95 ± 7.92 mmHg).

All subjects gave their written informed consent after receiving a detailed explanation of the study protocol. The design proposal, methods of data collection, and analysis of this study were approved by the ethics committee of the hospital.

### Image acquisition and analysis

Two-dimensional echocardiographic examinations were carried out with a GE Vingmed Vivid 7 (GE Vingmed Ultrasound, Horten, Norway) scanner equipped with a 1.7-3.4 MHz transducer (M3S probe). After a 15-minute rest in the supine position in a quiet room at 23°C, blood pressure (BP) and heart rate (HR) of all patients were measured three times and the mean values were calculated. An electrocardiogram (ECG) was also recorded simultaneously. The measurements and calculated formulas of the parameters in our study followed the 2015 American Society of Echocardiography and the European Association of Cardiovascular Imaging (ASE-EACVI) recommendations for chamber quantification.^13^ During ECG recording at a stable frame rate in the left lateral position, the RV end-diastolic diameter (RVED) was obtained in the middle third of RV inflow, approximately halfway between the maximal basal diameter and the apex, at the level of papillary muscles at end-diastole in the RV-focused apical four-chamber view with left ventricle (LV) apex at the center of the scanning sector; the RV anterior wall thickness (RVAW) was obtained below the tricuspid annulus, at a distance approximating the length of the anterior tricuspid leaflet in its fully open position and parallel to the RV free wall as seen from a subcostal four-chamber view. Both parameters were measured by a conventional, two-dimensional grayscale echocardiography.^13^ Tricuspid annulus plane systolic excursion (TAPSE) and peak systolic velocity of tricuspid annulus (S wave) were measured through the lateral portion of the tricuspid annulus by M-mode echocardiography and pulsed-wave tissue Doppler imaging (TDI) in the apical four-chamber view, respectively. RV fractional area change (RV FAC) was measured and calculated in the RV-focused apex four-chamber view: RV FAC (%) = 100 × (end-diastolic area [EDA] - end-systolic area [ESA]) / EDA.^13^ Three-dimensional echocardiographic RV ejection fraction (3D RV EF) was also measured: 3D RV EF (%) = 100 × (end-diastolic volume [EDV] - end-systolic volume [ESV]) / EDV.^13^ Left ventricular ejection fraction (LVEF) was measured by Simpson’s biplane method. PASP was estimated according to the simplified Bernoulli equation: PASP = 4×V^2^ (V = peak velocity of tricuspid regurgitation) + right atrial pressure (RAP). RAP was estimated through echocardiography based on the diameter and respiratory variation in diameter of the inferior vena cava (IVC). A diameter of IVC < 2.1 cm that collapses > 50% with a sniff suggests there is a normal RA pressure of 3 mmHg; while an IVC diameter > 2.1 cm that collapses < 50% with a sniff or < 20% on quiet inspiration suggests a high RAP of 15 mmHg; if the IVC diameter and collapse do not fit this paradigm, an intermediate value of 8 mmHg would be used.^14^

All images were digitally recorded in hard disks on offline analysis (EchoPAC version 8, GE Vingmed Ultrasound). Two-dimensional dynamic images were recorded for the subsequent analyses. A frame rate of 40-80 frames/s acquisition was used. All 2D-STE data were measured by averaging data of three heartbeats. We selected the most stable cardiac cycle for generation of the strain curve. After manually tracing the RV endocardium on apical four-chamber view, a region of interest (ROI) divided into six segments was automatically generated. Only RV free wall segmental strain was analyzed. Using a single frame from end-systole, the RV free wall segments were manually mapped by marking the endocardial border and the width of the myocardium. The parameters of ε and SRs, SRe and SRa were measured in RV free wall for basal, middle and apical segments, respectively, from the apical four-chamber view.

### Statistical analysis

The data were analyzed with SPSS 17.0 for Windows (SPSS, Chicago, IL, USA). Unpaired Student’s T-test was performed for continuous variables, which were all normally distributed. Numeric variables are presented as the mean ± standard deviation (SD). One-way analysis of variance (ANOVA) was performed to test for statistically significant differences among the four groups. Continuous data were compared between individual groups using the Student-Newman-Keuls post-test to test for statistically significant differences. All statistical tests were two-sided, and p < 0.05 was set for statistical significance.

## Results

### Patient characteristics

Between the four groups, there were no significant differences in age, sex, body mass index (BMI), body surface area (BSA), systolic blood pressure (SBP), and diastolic blood pressure (DBP). Nevertheless, the HR in group D was significantly higher than that in the other three groups (Seen in Table 1).

**Table 1:** Comparison of physiological parameters between systemic lupus erythematosus patients (groups B, C and D) and control group (Group A) (x ± *s*)

Parameters	Group A (n = 30)	Group B (n = 37)	Group C (n = 34)	Group D (n = 31)
Mean age, years	42.1 ± 10.50	45.3 ± 8.40	41.3 ± 9.60	43.3 ± 7.50
DBP, mm Hg	80.32 ± 3.66	79.92 ± 3.19	79.78 ± 4.97	82.52 ± 3.89
SBP, mm Hg	130.95 ± 5.27	128.4 ± 5.94	125.85 ± 9.07	128.39 ± 8.58
HR, beats/min	69.92 ± 9.57	73.13 ± 10.87	74.09 ± 8.61	89.52 ± 12.01^$*#^
BSA, m^2^	1.59 ± 0.26	1.67 ± 0.25	1.79 ± 0.38	1.66 ± 0.37
BMI, kg/ m^2^	26.38 ± 2.28	25.26 ± 2.94	25.56 ± 3.81	26.22 ± 1.46

DBP: diastolic blood pressure; SBP: systolic blood pressure; HR: heart rate; BSA: body surface area; BMI: body mass index.

$ p < 0.05 vs. group A

* p < 0.05 vs. group B

# p<0.05 vs. group C.

### Conventional echocardiographic parameters

There were no statistical differences in LVEF between the four groups. The RVAW and RVED were significantly higher in group D than in the other three groups, while TAPSE, RV FAC, pulsed Doppler S wave, and RV 3D EF were all significantly decreased in group D compared with the other groups. However, there were no significant differences in RVAW, RVED, TAPSE, RV FAC, pulsed Doppler S wave, and RV 3D EF between groups A, B and C (Seen in table 2).

**Table 2:** Comparison of conventional parameters between systemic lupus erythematosus patients (groups B, C and D) and control group (Group A) (x ± *s*)

Parameters	Group A (n = 30)	Group B (n = 37)	Group C (n = 34)	Group D (n = 31)	Reference normal value^@^
LVEF, %	64.51 ± 3.11	63.69 ± 6.61	62.11 ± 4.87	63.01 ± 4.86	≥ 50
RVAW, cm	0.36 ± 0.05	0.40 ± 0.03	0.43 ± 0.06	0.69 ± 0.09^$*#^	0.1-0.5
RVED, cm	2.98 ± 0.43	3.11 ± 0.45	3.22 ± 0.39	3.65 ± 0.36^$*#^	1.9-3.5
TAPSE, cm	2.24 ± 0.21	2.21 ± 0.19	1.76 ± 0.22	1.2 ± 0.18^$*#^	> 1.7
RV FAC, %	50.45 ± 4.67	49.24 ± 4.81	42.69 ± 5.07	34.43 ± 3.95^$*#^	> 35
Pulsed Doppler S wave, cm/s	13.35 ± 2.14	12.92 ± 1.90	11.48 ± 2.06	9.33 ± 1.81$*#	> 9.5
RV 3D EF, %	46.18 ± 2.28	45.80 ± 2.21	44.34 ± 2.14	31.19 ± 4.36$*#	≥ 40

LVEF: left ventricular ejection fraction; RVAW: right ventricular anterior wall thickness; RVED: right ventricular end-diastolic diameter; TAPSE: tricuspid annulus peak systolic excursion; RV FAC: right ventricular fractional area curve; Pulsed Doppler S wave: peak systolic velocity of tricuspid annulus by pulsed-wave tissue Doppler imaging; RV 3D EF: three-dimensional echocardiographic right ventricular ejection fraction.

$ p < 0.05 vs. group A

* p < 0.05 vs. group B

# p < 0.05 vs. group C. Chinese guidelines provide different reference normal values as compared with international guidelines.

### 2D-STE parameters

**Figure 1:**
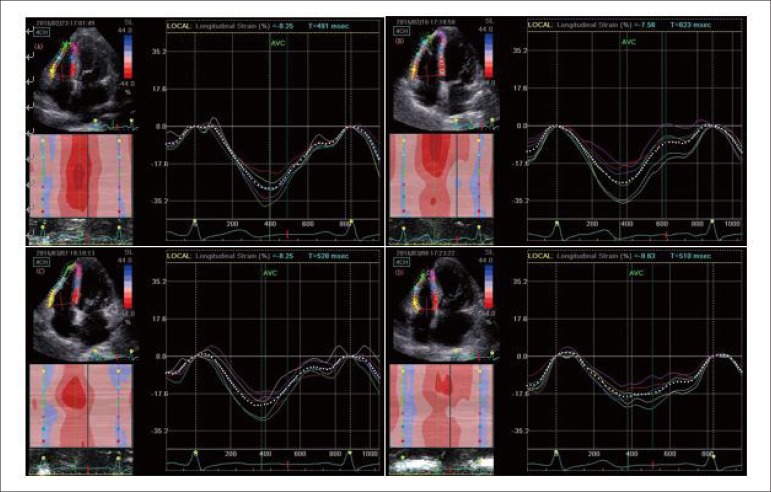
Longitudinal peak systolic strain (ε) curve was obtained in right ventricular free wall for basal, middle and apical segment by 2D-STE from the apical four-chamber view. (A) group A; (B) group B (systemic lupus erythematosus – SLE, without pulmonary hypertension); (C) group C (SLE with mild pulmonary hypertension); (D) group D (SLE with moderate-to-severe pulmonary hypertension).

The average of the longitudinal strain and SR of each segment in the basal, middle, and apical regions of the RV free wall was calculated in each group (Seen in table 3; Figure 1).

**Table 3:** Comparison parameters of strain rate and strain of the SLE patients with the control group (x ± *s*)

strain	Group A (n = 30)	Group B (n = 37)	Group C (n = 34)	Group D (n = 31)
**ε, %**				
Basal	-33.87 ± 5.89	-32.19 ± 7.38	-25.77 ± 7.67^↑*^	-19.55 ± 4.89^$*#^
Middle	-31.67 ± 7.00	-29.09 ± 7.30	-22.89 ± 8.05^↑*^	-17.67 ± 6.83^$*#^
Apical	-25.45 ± 6.99	-27.51 ± 2.47	-19.64 ± 8.65^↑*^	-15.91 ± 6.33^$*#^
**SRs** **, s^-1^**				
Basal	-2.33 ± 0.34	-2.43 ± 0.44	-1.84 ± 0.41^↑*^	-1.73 ± 0.47^$*^
Middle	-1.78 ± 0.34	-1.67 ± 0.56	-1.59 ± 0.37^↑*^	-1.36 ± 0.31^$*^
Apical	-1.53 ± 0.54	-1.54 ± 0.55	-1.33 ± 0.38^↑*^	-1.16 ± 0.36^$*^
**SRe** **, s^-1^**				
Basal	2.44 ± 0.74	2.43 ± 0.69	1.95 ± 0.49^↑*^	1.85 ± 0.52^$*^
Middle	2.04 ± 0.58	2.06 ± 0.49	1.73 ± 0.54^↑*^	1.66 ± 0.46^$*^
Apical	1.84 ± 0.69	1.83 ± 0.67	1.33 ± 0.65^↑*^	1.29 ± 0.55^$*^
**SRa, s^-1^**				
Basal	1.66 ± 0.64	1.63 ± 0.66	1.44 ± 0.56^↑*^	1.42 ± 0.55^$*^
Middle	1.55 ± 0.70	1.56 ± 0.65	1.28 ± 0.41^↑*^	1.21 ± 0.52^$*^
Apical	1.88 ± 0.49	1.85 ± 0.67	1.60 ± 0.56^↑*^	1.54 ± 0.54^$*^

SRs: systolic strain rate; SRe: early diastolic strain rate; SRa: late diastolic strain rate

$ p  <0.05 vs. group A

* p < 0.05 vs. group B

# p < 0.05 vs. group C.

We found that there were no significant differences in all the parameters between groups A and B. On the other hand, in groups C and D, ε, SRs, SRe and SRa of each segment were significantly decreased compared with groups A and B. The parameter ε of each segment in group D was also significantly lower than that in group C, although there were no significant differences in SRs, SRe and SRa of each segment between groups C and D.

## Discussion

It has been previously demonstrated that RV function is a decisive factor for the severity and prognosis of SLE patients with PH,^15^ and that 2D-STE-derived strain and strain rate imaging could precisely reflect deformation of RV myocardium, and detect the subclinical RV dysfunction.^16^ Thereby, evaluation of RV function in SLE patients with PH is important for establishing treatment strategy, prevent clinical RV dysfunction and RV failure, and increase the survival rate of SLE patients with PH. To our knowledge, this has not been studied before.

In the present study, we found that there were no significant differences in age, sex, BMI, BSA, SBP, and DBP between the four groups. Nevertheless, the HR in group D was significantly higher than that in the other three groups. It has been reported that HR could affect ε, and the increased HR was related to reduced ε, which represents the degree of deformation.^17-20^ It also indicates that the degree of deformation of group D was decreased.

The function of the RV is to maintain the normal blood flow of pulmonary circulation, which mainly depends on three factors: preload, contraction, and afterload.^21^ PH is a common and devastating complication of SLE characterized by progressively increased pulmonary vascular resistance (PVR) and PASP.^22^ Its mechanism is very complex and closely related to inflammation and the immune system.^23,24^

In this study, we found that RVAW and RVED were significantly higher in group D than those in the other three groups, while TAPSE, RV FAC, pulsed Doppler S wave, and RV 3D EF were all significantly decreased in group D compared with the other groups. However, there were no significant differences in RVAW, RVED, TAPSE, RV FAC, pulsed Doppler S wave, and RV 3D EF between groups A, B and C. It demonstrates that the structure of the RV was remodeled in group D, and the RV myocardial systolic function was also impaired. We argue that long-standing increases in PASP in SLE patients with PH cause increased RV afterload, decreased pulmonary vascular compliance, and compensatory increases in RV contractility. Structurally, these results in expansion of the right ventricle and increased RV wall thickness for maintenance of RV function.^18,22,25^ As PASP further increases, the impaired RV myocardium undergoes hypoxia, which causes enlarged RV volume, tricuspid valve insufficiency, and increased RV preload. This progresses to increased right atrial diameter and exacerbated myocardial impairment, leading to RV remodeling and decompensation, reduced RV contraction, and finally clinical RV dysfunction.^22,26^ Based on conventional data, group D experienced clinical RV dysfunction. Decreased TAPSE, RV FAC and pulsed Doppler S wave also implied a bad prognosis, and decreased RV 3D EF even triggered the RV failure of patients in group D, while the RV function in group C was still normal.

In this prospective study, based on the 2D-STE data, we found that ε, SRs, SRe and SRa of each segment were significantly decreased in groups C and D compared with groups A and B, while there were no significant differences in these parameters between groups A and B. The parameter ε of each segment in group D was also significantly lower than that in group C, although there were no significant differences in SRs, SRe and SRa of each segment between groups C and D. As mentioned before, ε represents the degree of deformation, and SR represents ventricular contractility.^27^ This means that the degrees of RV deformation in groups C and D were significantly lower compared with groups A and B, and significantly lower in group D than in group C. This implies that the RV function of both groups C and D was impaired, and this was more severe in group D. This is in accordance with the findings of Pirat et al.,^28^ The discrepancy of ε and SR between groups C and D in this study might be related to the significant differences in HR and PASP in these groups. While SR has been shown to be independent of load, HR and other factors, an increased HR and altered load changes have been associated with reduced ε.^17-20^ The impaired RV function of group C (mild PH group) was detected early by 2D-STE-derived strain and SR imaging compared with the conventional echocardiography.

### Study limitations

There were several limitations of this study. First, PH was not determined via right heart catheterization, but estimated by echocardiography. Second, RV was not assessed by cardiac magnetic resonance for the sake of comparison. However, these might not be a limitation of this study, because it aimed to evaluate and compare the RV function of SLE patients with different degrees of PH estimated by 2D-STE-derived strain and SR imaging. Third, clinical data of SLE patients, such as antiphospholipid antibodies were not collected. Also, estimation of PH by 2D-STE may be influenced by factors, such as patient’s breathing pattern. Finally, some participants such as obese patients may not be able to use this method, because it requires high-resolution image quality.

## Conclusion

In conclusion, 2D-STE-derived strain and SR imaging could early detect the RV dysfunction in SLE patients with PH, especially in those with mild PH. This has an important value in guiding early therapy in clinical settings, improving the prognosis, and increasing the quality of life of SLE patients with PH.
